# Endoscopic Botulinum Therapy for Obesity: Focus on the Antrum and Fundus

**DOI:** 10.7759/cureus.87319

**Published:** 2025-07-05

**Authors:** Kodai Takahashi

**Affiliations:** 1 Surgery, Tokyo Takahashi Clinic Nerima, Tokyo, JPN

**Keywords:** antrum, endoscopic injection, fundus, gastric botulinum therapy, non-surgical weight loss, obesity treatment, whole-stomach injection

## Abstract

Introduction: Gastric botulinum toxin therapy is gaining attention as a non-invasive treatment for obesity. However, existing studies show inconsistent results and standardized protocols remain lacking. We aimed to evaluate the efficacy and safety of a novel whole-stomach injection method emphasizing the antrum and fundus in obese patients in Japan.

Methods: A total of 144 obese patients (mean age 42.3 years, mean BMI 30.3 kg/m²) underwent gastric botulinum toxin therapy at our institution between February 2023 and November 2024. Coretox (300-400 U) was injected endoscopically across the entire stomach, with increased concentration in the antrum and fundus. Patients were followed for six months post procedure. The primary outcome was the percentage of total body weight loss (%TBWL), and safety was assessed by the occurrence of complications per Clavien-Dindo classification.

Results: Mean %TBWL was 6.5% at one month, 10.2% at three months, and 12.2% at six months post treatment. No complications or adverse events were reported. The average procedure time was 5.7 minutes, and all patients completed follow-up as scheduled. We hypothesize that the observed efficacy may be partially attributed to our refined injection technique and individualized BMI-based dosage adjustment, although further controlled studies are needed to validate this approach.

Conclusion: Gastric botulinum therapy using a novel full-stomach injection method focusing on the antrum and fundus was found to be both safe and effective in a Japanese obese population. This technique may offer a promising, minimally invasive option for obesity management. Further multicenter and long-term studies are warranted to validate and standardize this approach.

## Introduction

Obesity is a major risk factor for lifestyle-related diseases such as diabetes, hypertension, and cardiovascular disorders, posing serious health and social challenges. With a global increase in the obese population, the associated medical and economic burdens have also been expanding. In Japan, obesity is defined as a BMI of ≥25 kg/m². According to the National Health and Nutrition Survey of Japan (2021), approximately 33.0% of adult men and 22.3% of adult women are classified as obese (BMI ≥25 kg/m²). While dietary therapy and exercise therapy are recommended as primary treatments for obesity management, there remain patients who do not respond to these therapies and those who cannot opt for pharmacological or surgical interventions. Hence, there is a growing need for new therapeutic approaches [1-2].

Botulinum toxin has recently gained attention as a non-surgical obesity treatment due to its ability to suppress gastric motility and delay gastric emptying. This pharmacological effect may enhance satiety and reduce caloric intake, as shown in several preliminary clinical studies [3-5]. 

Past studies, mainly from Western countries, have reported on the efficacy of gastric botulinum therapy. However, the treatment outcomes have been inconsistent, and the type of botulinum toxin, injection sites, and doses used are not standardized, posing challenges [3-5]. Moreover, there are limited studies targeting Asian populations, and data on efficacy and safety in these patients remain insufficient [4].

This study aimed to provide new clinical evidence on the effects and safety of gastric botulinum toxin therapy using a novel injection protocol that distributes the toxin across the entire stomach, with a focused emphasis on the antrum and fundus, an approach designed to enhance satiety and delay gastric emptying more effectively than antral-only methods used in prior studies. Furthermore, this single-center experience contributes to standardizing this therapeutic strategy within the Japanese population.

## Materials and methods

Patient selection

A total of 144 obese patients who underwent gastric botulinum therapy at our hospital between February 2023 and November 2024 were included in this study. This study is a retrospective analysis of obese patients who underwent whole-stomach botulinum injections at our clinic. The following treatment protocol was used. The mean age of the patients in this study was 42.3 years, and their mean BMI was 30.3 kg/m². All patients had a BMI of ≥25 kg/m² (Table 1).

**Table 1:** Demographic and clinical characteristics of the patients

Characteristics	Value
Number of patients	144
Age (years), mean ± SD	42.3 ± 11.5
Gender, n (%)	
Male	62 (43.1%)
Female	82 (56.9%)
Baseline BMI (kg/m²), mean ± SD	30.3 ± 4.8
BMI ≥ 30 kg/m², n (%)	91 (63.2%)
Procedure time (min), mean ± SD	5.7 ± 1.2
Botulinum toxin dose, n (%)	
300 U (BMI 25–29.9 kg/m²)	53 (36.8%)
400 U (BMI ≥30 kg/m²)	91 (63.2%)
Adverse events, n	0
Completion rate, n (%)	144 (100%)

Eligible patients were defined as those with a BMI > 25 kg/m² who had obesity-related comorbidities and had failed to achieve weight loss despite prior dietary and exercise therapy. Failure of dietary and exercise therapy was defined as less than 5% total body weight loss over a minimum of six months of supervised lifestyle intervention. Comorbidities such as diabetes mellitus, hypertension, and dyslipidemia were diagnosed based on medical records and physician-confirmed diagnoses according to Japanese clinical guidelines. Exclusion criteria included pregnancy, severe cardiac disease, liver or renal disease, active cancer, and any history of hypersensitivity reactions. All patients provided written informed consent. This study was approved by the Institutional Review Board of Tokyo Takahashi Clinic Nerima, Tokyo, Japan (IRB No. 2023-01). The present study was approved by the Institutional Review Board to ensure the protection of patient privacy and confidentiality and was undertaken in accordance with the ethical standards of the World Medical Association Declaration of Helsinki.

Anesthesia and procedure

All treatments were performed under intravenous anesthesia using propofol.

Medication and dosage

The amount of botulinum toxin administered was adjusted according to the patient’s BMI. Specifically, patients with a BMI of ≥25 kg/m^2^ received 300 units (U) of Coretox, and those with a BMI of ≥30 kg/m^2^ received 400 U of Coretox. Botulinum toxin was reconstituted with 30 mL of preservative-free normal saline for the 300 U dose and 40 mL for the 400 U dose.

Injection technique

The following injection technique, originally developed by the authors for this study, was used to achieve uniform distribution of botulinum toxin throughout the stomach. First, 1 mL of botulinum toxin was injected in four quadrants at a point 2 cm proximal to the pyloric ring. Additional 1 mL injections were then performed in a systematic manner every 4 cm along the greater curvature, extending from the distal antrum to the gastric cardia. In total, injections were administered at approximately 30-40 sites, depending on individual gastric length.

This technique was specifically designed to ensure full stomach coverage, including the antrum, corpus, and fundus, with proportional dosing adjusted to gastric anatomy. The intent was to enhance the delay of gastric emptying and suppress ghrelin secretion by targeting both motility and hormonal axes. To our knowledge, this whole-stomach injection method has not been previously described in the literature and represents a novel protocol devised in the context of clinical practice and anatomical optimization.

Follow-up

Follow-up evaluations were conducted at approximately one month, three months, and six months post treatment. Adverse events were actively monitored during scheduled follow-up visits and through interim phone interviews conducted by clinic staff. The severity of each postoperative complication was evaluated using the Clavien-Dindo Classification: grade 0, no complication; grade I, deviation from the normal postoperative course without the need for therapy; grade II, complications requiring pharmacological treatment, such as antibiotic administration; grade III, complications requiring endoscopic, radiological, or surgical intervention; grade IV, life-threatening complications requiring intensive care; and grade V, death.

The primary outcome of this study was the percentage of total body weight loss (%TBWL) at six months following the procedure.

Statistical analysis

Statistical analysis was performed using JMP® 11 (SAS Institute, Cary, NC, USA). Data normality was assessed using the Shapiro-Wilk test. For comparisons across time points, repeated-measures ANOVA was used for normally distributed data and the Friedman test for non-parametric data. Post hoc pairwise comparisons were conducted using the Wilcoxon signed-rank test. Subgroup analyses were performed by gender and baseline BMI category. Statistical significance was defined as p < 0.05. Repeated-measures ANOVA was used for the longitudinal comparison of normally distributed %TBWL data; for non-parametric distributions, the Friedman test was applied. Post hoc pairwise comparisons, where appropriate, were conducted using the Wilcoxon signed-rank test.

## Results

Patient demographics and procedural outcomes

A total of 144 patients underwent whole-stomach botulinum toxin injection therapy with targeted emphasis on the antrum and fundus. The cohort comprised 82 females (56.9%) and 62 males, with a mean age of 41.7 ± 11.5 years and a mean baseline BMI of 31.4 ± 4.8 kg/m². The procedure was technically successful in all cases, and no intraoperative or delayed complications were reported. The mean procedure time was 5.7 ± 1.2 minutes (range: 4.2-8.1 minutes), reflecting high procedural efficiency suitable for outpatient day-surgery settings.

Weight loss outcomes

Weight loss outcomes were favorable. The mean %TBWL at each time point was as follows: one month: 6.5% ± 2.1% (interquartile range (IQR): 5.2-7.8%); three months: 10.2% ± 3.4% (IQR: 8.0-12.4%); six months: 12.2% ± 4.2% (IQR: 9.5-14.6%)

**Figure 1:**
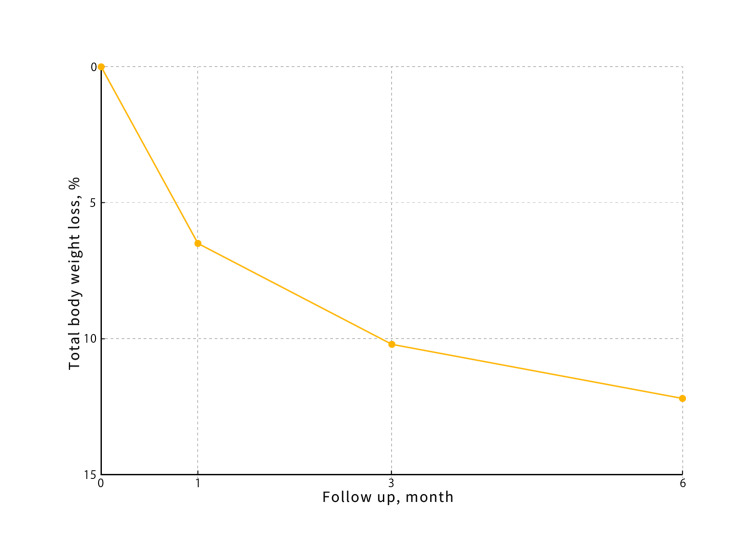
Percentage of total body weight loss after gastric botulinum therapy

Figure 1 shows %TBWL at one, three, and six months post procedure, presented as mean ± standard deviation with error bars and corresponding n values.

At six months, patients with a baseline BMI ≥35 kg/m² achieved a %TBWL of 13.8% ± 3.9% (IQR: 11.2-16.4%), compared to 11.1% ± 4.1% (IQR: 8.6-13.7%) in those with BMI <35 kg/m². Although this difference did not reach statistical significance (p = 0.07), it may suggest a trend toward a BMI-dependent treatment response.

Additionally, at the six-month follow-up, 78.5% (113/144) of patients achieved greater than 10% TBWL, a commonly used benchmark for clinically meaningful weight loss.

Safety and tolerability

All 144 patients completed the treatment protocol, including scheduled follow-ups at one, three, and six months. No adverse events requiring clinical intervention were reported. Patients were monitored through in-person visits and telephone follow-ups at one, three, and six months post procedure. The assessment included symptoms such as nausea, abdominal pain, and signs of delayed gastric emptying. No hospitalizations or emergency interventions were required.

Patient-reported outcomes

Treatment tolerability was assessed using a five-point Likert scale questionnaire administered at the one-week post-treatment follow-up. Although the questionnaire was not formally validated, it was developed based on clinical experience. Overall, 96.5% of patients rated their experience as either “excellent” or “good.”

## Discussion

Treatment safety and tolerability

In this study of 144 cases of gastric botulinum therapy at our high-volume center, treatment safety was confirmed. Specifically, there were 0 complications during or after the procedure, and no adverse events were reported. Our findings suggest that gastric botulinum injection appears safe and well-tolerated in the short term within this cohort. Moreover, adjusting the injection volume according to each patient’s BMI and gastric size, as well as distributing the toxin evenly throughout the stomach, likely contributed to the consistency of the treatment effect.

Ideally, it would be best to perform an endoscopy beforehand to determine gastric size and then decide the dose. However, many patients refused preoperative endoscopy, making this difficult to implement. According to previous literature, abdominal pain, nausea, and bloating are the main side effects of this therapy. Generally, these side effects are mild to moderate and tend to resolve spontaneously, with an incidence of approximately 10% to 20% and serious complications being rare [5]. The present study suggests that the outcomes and side effects of botulinum therapy may depend on factors such as the type of botulinum toxin used and the dose administered.

Efficacy and comparative literature

Regarding efficacy, the %TBWL at six months post treatment was 12.2%, which is comparable to results reported by other studies [6-12]. All patients completed the treatment plan without complications, confirming a very high success rate. Additionally, tolerability was good in all patients, indicating that the burden on patients is minimal.

In previous literature, many studies have focused primarily on injections into the pylorus. However, injections only into the pylorus may result in reduced efficacy [6]. While giving priority to pyloric injections, appropriately administering injections into the greater curvature and the fundus, where ghrelin is secreted, may be a key factor in enhancing treatment efficacy.

A systematic review and meta-analysis conducted in 2020 showed that gastric botulinum therapy significantly reduced body weight in patients with a BMI ≥40 kg/m², while similar effects were not observed in those with a BMI <40 kg/m². On the other hand, the 2018 randomized controlled trial (RCT) found that endoscopic ultrasound-guided gastric botulinum therapy in patients with severe obesity did not demonstrate significant weight loss compared to placebo, indicating limitations in its effectiveness [12]. A 2023 study concluded that intragastric botulinum toxin A has limited efficacy for weight loss, with modest benefits seen only in patients with a BMI >40 kg/m² when high-dose, multi-site injections are combined with dietary control [13]. These findings collectively indicate that the effect of gastric botulinum therapy is highly dependent on factors such as patient BMI, adjunctive therapies, and treatment conditions.

Additionally, the study by Kanlioz et al. discussed the influence of pyloric tone on treatment success. They reported a significant decrease in BMI among patients with normal-toned pylori (NP), whereas no effect was observed in hypotonic pylori [14]. This suggests that assessing pyloric function when selecting patients may be crucial for enhancing treatment efficacy.

Injection strategy: anatomical rationale

Although gastric botulinum therapy is gaining attention as a new alternative for obesity treatment, its efficacy has not been consistently demonstrated in the literature [6-9]. A previous study performed a single-site injection at the pylorus without dietary guidance and found no significant TBWL effect, highlighting the possible role of injection distribution and diet [6]. Several RCTs have suggested that gastric botulinum therapy can delay gastric emptying and aid in weight loss; however, its effectiveness varies by patient BMI and concurrent treatments [9-11].

Compared to prior studies employing antral-only or fundus-only botulinum injection, the whole-stomach approach in our study resulted in a higher %TBWL, suggesting a potential synergistic effect when both regions are targeted simultaneously. This broader distribution may enhance the suppression of gastric motility and appetite-regulating hormones, particularly ghrelin, thereby improving weight loss outcomes. These findings support the notion that injection strategy, including anatomical targeting, plays a crucial role in therapeutic efficacy [6-11].

In the present study, it is speculated that adjusting the dose according to the patient’s BMI and gastric size and distributing the botulinum toxin evenly throughout the stomach contributed to the favorable outcomes observed. Moreover, precise adjustments of the injection sites might have effectively impacted gastric contractions and hormone secretion, contributing to consistent results.

Hormonal mechanisms and ghrelin suppression

The potential impact of gastric botulinum therapy on ghrelin secretion, a hormone that regulates appetite, has drawn attention [15-17]. Several mechanisms have been proposed by which botulinum toxin might affect ghrelin. First, botulinum toxin could indirectly decrease ghrelin secretion by inhibiting the nerve and muscle activity of the stomach. Second, by prolonging gastric retention time, botulinum toxin could lengthen the sensation of satiety and potentially suppress ghrelin secretion. Although ghrelin modulation is a plausible mechanism based on prior studies [15-17], our study did not assess hormonal levels directly. Thus, this remains speculative. Nevertheless, gastric botulinum therapy holds promise as a novel approach aimed at improving appetite regulation and energy metabolism through the ghrelin pathway.

Limitations and generalizability

While this study demonstrates promising results regarding the safety and short-term efficacy of whole-stomach gastric botulinum toxin injection, several limitations must be acknowledged. First, this was a retrospective single-center study without a control group, which limits the ability to infer causal relationships and generalize the findings. Second, the follow-up period was limited to six months, and longer-term outcomes, including weight maintenance and potential metabolic improvements, were not evaluated. Third, although the procedure was standardized and performed at a high-volume center, individual differences in gastric anatomy and injection depth may have introduced variability in the treatment effect. Fourth, we did not evaluate changes in gastric emptying time, ghrelin levels, or other hormonal biomarkers, which could provide mechanistic insights into the observed weight loss. Fifth, participants were selected based on their willingness to undergo this novel therapy without prior endoscopy, introducing possible selection bias. Finally, all participants were Japanese, and the generalizability of the results to other populations with differing dietary patterns, BMI distributions, or gastric morphology remains unclear.

Future directions

When considering the efficacy of gastric botulinum therapy for Japanese patients with obesity, it is necessary to take into account cultural and lifestyle factors (e.g., body shape and dietary habits) and compare findings with Western data. In this study, because the patients were relatively young (mean age 42.3 years) and had moderately high BMI (mean 30.3 kg/m^2^), we only evaluated weight loss and safety, without analyzing improvements in obesity-related complications. Moreover, to assess the long-term safety, weight reduction, and improvements in obesity-related complications, further investigations such as multicenter collaborative studies or RCTs are needed.

A limitation of gastric botulinum therapy is that its effect may be transient and applicable to only certain patient groups. However, key advantages include its minimally invasive nature, the possibility of repeated administration every six months without the development of drug resistance (unlike pharmacotherapy), and its utility for patients who are ineligible for surgical interventions. Regarding repeatability, previous reports and our experience suggest that reinjection after six months is feasible in some patients. Regarding cost-effectiveness, although not formally analyzed, the therapy’s simplicity and non-reliance on expensive surgical infrastructure may offer an economic advantage.

Unfortunately, patient satisfaction data were not systematically collected in this study and therefore cannot be compared with previous literature. Future research should aim to identify predictive factors for treatment efficacy, considering patient characteristics such as age, gender, comorbidities, and BMI. With further research, this treatment may become more firmly established as a therapeutic option in clinical practice.

## Conclusions

This study demonstrates that tailoring the dose of botulinum toxin and refining the injection technique according to individual patient characteristics are critical for enhancing both the efficacy and safety of gastric botulinum therapy. Future research is expected to further standardize these treatment techniques, ultimately promoting their broader clinical application.
